# Regaining the path to malaria elimination: Lessons from the pandemic

**DOI:** 10.5281/zenodo.10948595

**Published:** 2024-04-09

**Authors:** Michael Macdonald

**Affiliations:** 1. Catonsville, Maryland, USA.

## Abstract

The stagnation in malaria elimination efforts can be attributed to several contributing reasons: large populations displaced by conflict and severe weather, insecticide and drug resistance, competing priorities with COVID-19 and Ebola. Part of the problem may also be us and our pre-pandemic systems. The accelerated response to the COVID-19 emergency carries lessons for global efforts against the ‘other emergency’, malaria. Michael has worked in vector control since 1977, beginning with Peace Corps in the Sabah (E. Malaysia) MCP. He earned an Sc.D. from Johns Hopkins researching malaria transmission in Pakistan; lived in Burma, Thailand, Cambodia and Zambia with stints in the US and Geneva supporting programmes throughout Africa and Asia, working for Johns Hopkins and Boston Universities, USAID, WFP, UNHCR, WHO, IVCC and NGOs involved in public health entomology and vector control in Africa and Asia.

Since 2015, gains in global malaria control efforts have stalled. After significant progress in lives-saved since the start of the millennium with increased funding to expand access to insecticide-treated bednets, indoor residual spraying, new diagnostics and treatments, we are at a crossroads and no longer on track to meet our malaria elimination goals.

There are several contributing reasons, large populations displaced by conflict and severe weather, insecticide and drug resistance, competing priorities with COVID-19 and Ebola. Part of the problem may also be us and our pre-pandemic systems of more recent years since Global Fund enabled resources to flow along very narrow pathways proscribed in Geneva.

Arundhati Roy published “The Pandemic as Portal” in the Financial Times, April 3, 2020, where she wrote:

*Historically, pandemics have forced humans to break with the past and imagine their world anew. This one is no different. It is a portal, a gateway between one world and the next. We can choose to walk through it, dragging the carcasses of our prejudice… our data banks and dead ideas. Or we can walk through lightly, with little luggage, ready to imagine another world. And ready to fight for it.* [1]

The accelerated response to the emergency, with new coronavirus diagnostics, vaccines and therapeutics, as well as the intense healthcare system response, carry lessons for global malaria efforts. The public sector commitment to work in partnership with the private sector to develop these technologies and expedite their review, country registration and deployment stands in stark contrast to the way we approach “the other emergency” of malaria.

## Industry is not ‘otherness’

We old malariologists often hold the commercial sector at arm’s length, ‘vendors’ rather than ‘partners’ competing in massive global tenders for the lowest possible unit cost. With insecticide-treated bednets, this cost drive to the bottom stifles innovation for durability quality improvement, user acceptability and incorporation of new chemistries to combat insecticide resistance.

In a discussion with a former director of the WHO Global Malaria Programme, about the prohibition of industry partners being invited to a WHO meeting on insecticide resistance for fear they are looking only for their own self-interest, I remarked:

*“We’re like an old married couple. We’re no longer trying to woo each other, but we have some problem children we need to deal with.”*

And is the self-interest of industry any different than the self-interest of researchers looking to advance their own careers and institutions demanding yet more funding for studies before product approval? We had several examples of public private partnerships for improved access to public health commodities in the WASH sector, reproductive health as well as malaria vector control before the massive funding available in the early 2000s allowed us to ignore those partnerships and try to go it alone [2].

## Expedited approvals (Not): Beyond the “Gold Standard” RCT with epidemiological outcomes

The approval process for malaria products is numbingly slow; taking years for innovations that may have already been approved for safety and efficacy by stringent regulatory authorities such as the US Environmental Protection Agency. There are often demands for prohibitively expensive Randomised Control Trials (RCTs) with epidemiological endpoints. While RCTs are the ‘gold-standard’ for therapeutics and vaccines, they may be inappropriate and at times unethical in context-specific environmentally driven processes like malaria transmission. There is an old joke that “RCT is the Gold Standard is like a shiny rock that only has value because people with a vested interest say so”.

The demand for RCTs with epidemiological endpoints, versus entomological endpoints and modelling has a direct impact on hindering tool development for low-prevalence settings, such as forest-goers in the Greater Mekong Subregion, where powering a significant trial is prohibitively expensive. Similarly, RCTs with epidemiological endpoints may not be ethical for trials in humanitarian emergencies. But as pointed out for housing improvement and malaria, absence of this ‘gold standard’ prevents global policy and significant donor funding. [3]

We can learn from the WASH sector, who are moving away from RCTs for water, sanitation and hygiene project evaluation based on epidemiological outcomes such as stunting and diarrhoeal disease, into 'Transformative WASH’, beginning with Human Centred Design, stakeholder alignment, tailored evaluation designs, iterative programme learning and scale-up, discovering not just what works, but why things work [4]. In vector control we can move away from prohibitive epidemiological endpoints to prove ‘Public Health Value’ similar to the WASH sector, by improved entomological endpoints and vectorial capacity [5], modelling, Human Centered Design [6] and soon, serological markers for vector exposure [7].

## Global leadership: Solution implementing versus problem solving; guidelines or capacity?

If we are to succeed in our malaria elimination efforts, we need to remember the words of Dr. José Nájera, former director of the WHO malaria program:

*“Before DDT, malariologists were trained as problems solvers, after DDT, malariologists were trained as solution implementors”.* [8]

Unfortunately, current global leadership seems entirely focused on ‘guidelines’ and edicts to donors and programmes on exactly what’s allowed in implementing a very narrow set of interventions. In 2013, WHO initiated a framework for capacity building for public health entomology, that we thought at that time was at a ‘crossroads’ but that initiative was allowed to fade [9].

Bruce Springsteen’s interview with President Obama discussed leadership styles, the top-down command and control, versus bottom-up capacity building [10]. As Obama was quoted earlier:

*“Anyone who wants to be a leader, ask yourself ‘How am I helping other people do great things’. Give them the tools and get rid of the barriers and help coach them so they can do a great job.”* [11].

The WHO Global Malaria Programme needs to reconsider priorities from generating yet more RCTs to building a new generation of problem-solvers with the flexibility and the resources they need with this new mantra ‘sub-national tailoring’.

A favourite example of leadership removing barriers and providing flexibility to staff to ‘solve the problem’ comes from Sri Lanka and the entomologist, the late Ms Lalanthika Peiris. Her story was related to me by Prof. Kamini Mendis and paraphrased here:

2009 marked the end of the civil war, and when the incidence of malaria in the country was brought down to very low levels, clearly on the way to elimination, there was a sudden increase in malaria attributable to Hambantota. Lalanthi found the outbreak was due to a series of army camps located by the Menik river, caused by soldiers returning from conflict areas of the north where malaria was still prevalent. *Anopheles culicifacies* was breeding in large numbers in the drying up river which then sparked local transmission. Lalanthi obtained the cooperation of the army officers in charge of the camps and mounted a massive malaria control operation by larviciding several kilometers of the river – a task that may have seemed impossible to most, and may have even contravened the standard guidelines for malaria control. In just over a year, they quelled the outbreak completely, by early 2011, and Sri Lanka eliminated malaria in 2012 (and was certified by WHO as malaria-free in 2016). If that *Plasmodium vivax* outbreak had not been eliminated rapidly, the disease would have almost certainly been carried to other parts of the country by army personnel travelling home and other duty stations throughout Sri Lanka and very likely malaria would still be prevalent there today.

Ms. Peiris had the flexibility and empowerment and ‘may have even contravened the standard guidelines for malaria control’ thus preventing local transmission and enabling Sri Lanka to continue on the path to malaria elimination.

A second example of unsung heroes who through their own dedication to malaria and their communities, solving problems, not implementing solutions is Mr. Yeang Chheang from Cambodia. Starting with the Malaria Eradication Programme in 1955, surviving the genocidal Pol Pot years, he helped restart the programme in 1979 with only 12 of the previous 400 staff that could be located, introduced ITNs, started the National Dengue Control Programme and put the Cambodia programme on the path to near elimination. Mr. Yeang Chheang was honoured at the December 2023 Health Day at COP 28 in Dubai for his remarkable devotion to malaria. A video of his award is available [12].

These are the heroes Dr. Nájera acknowledges. They had the capacity and initiative to ‘solve the problem’, and not simply ‘implement the solution’ that took years of strict policy development while their communities waited.

## Humanitarian emergencies

The RBM Vector Control Working Group’s work-stream on vector control in humanitarian emergencies [13] mission statement reads:

- improve delivery, uptake, integration and evaluation of existing vector surveillance and control tools;
- facilitate the development of an evidence-base and uptake of supplementary and emerging tools.

The workstream issued a statement on World Mosquito Day, 20 August 2023, noting that forcibly displaced persons now total more than 110 million; many are at increased risk for vector-borne diseases: malaria throughout much of Africa, leishmaniasis in Syria, Turkey and Iraq, dengue in Yemen and Bangladesh, among others. While some are in camps or settlements where standard tools can be deployed, many others are mobile, in makeshift shelters and situations where these tools are not practical.

There is a gap where new tools in development could play a life-saving role if WHO would provide ‘emergency use listing’, as it did in response to COVID-19, that would enable donor funding for large-scale pilot deployment. We will not reach global malaria targets unless we address these ever-expanding challenges, especially during the acute phase of humanitarian emergencies [14].

Vector control in the acute aftermath of an emergency or disaster is NOT traditional vector control. It must not circumscribe interventions framed by ‘traditional’ vector control as proscribed by VCAG but address the broad spectrum of pests and vectors caused by crowding, inadequate housing and sanitation, environmental challenges and disruption of community systems. We need to fast-track development and deployment, learning by doing, providing emergency-use authorisation for emerging vector control tools including spatial repellents, IRS adapted for temporary shelters, etofenprox-treated materials, improved targeting and delivery of larvicides – all at an advanced stage of development, just waiting for word from Geneva.

As I began this essay, ‘The Pandemic as Portal’. We hope now, in 2024, nearly a decade into the stagnation of our malaria control efforts, we heed Arundhati Roy, break from the past, learn the lessons from COVID-19, change our systems and imagine a new path towards a malaria-free world.
